# The Application of Black Phosphorus Nanomaterials in Bone Tissue Engineering

**DOI:** 10.3390/pharmaceutics14122634

**Published:** 2022-11-28

**Authors:** Xirui Jing, Zekang Xiong, Zian Lin, Tingfang Sun

**Affiliations:** 1 Department of Orthopedics, Union Hospital, Tongji Medical College, Huazhong University of Science and Technology, Wuhan 430022, China; 2 Hubei Key Laboratory of Bioinorganic Chemistry and Materia Medica, School of Chemistry and Chemical Engineering, Huazhong University of Science and Technology, Wuhan 430022, China

**Keywords:** black phosphorus, bone, 2D nanomaterials, bone regeneration, bone tissue engineering

## Abstract

Recently, research on and the application of nanomaterials such as graphene, carbon nanotubes, and metal–organic frameworks has become increasingly popular in tissue engineering. In 2014, a two-dimensional sheet of black phosphorus (BP) was isolated from massive BP crystals. Since then, BP has attracted significant attention as an emerging nanomaterial. BP possesses many advantages such as light responsiveness, electrical conductivity, degradability, and good biocompatibility. Thus, it has broad prospects in biomedical applications. Moreover, BP is composed of phosphorus, which is a key bone tissue component with good biocompatibility and osteogenic repair ability. Thereby, BP exhibits excellent advantages for application in bone tissue engineering. In this review, the structure and the physical and chemical properties of BP are described. In addition, the current applications of BP in bone tissue engineering are reviewed to aid the future research and application of BP.

## 1. Introduction

Phosphorus is a highly abundant element in the human body and accounts for approximately 1% of the total body weight [1]. Phosphorus plays various important physiological roles in the body. For example, it participates in the composition of nucleic acids, ATP, and other substances, and it maintains homeostasis in the body’s internal environment [2,3]. Most of the phosphorus in the human body exists in bone tissue in the form of hydroxyapatite, which is used to maintain the mechanical strength of bone tissue [4]. Phosphorus plays an essential role in promoting osteogenic repair during bone repair owing to its significant proportion in bone tissue [5].

Black phosphorus (BP) is a novel nanomaterial composed of phosphorus monomers. It has attracted the attention of researchers since the first report of two-dimensional (2D) BP nanosheets (BPNs) in 2014 [6]. BPNs consist of a unique layered structure of stacked phosphorus elements connected by van der Waals forces. This weak interaction enables BP to conveniently peel off the surface of the bulk BP crystal through different peeling modes [7]. BP is more stable than other phosphorus monomers (red and white phosphorus) that are non-flammable at room temperature and insoluble in most solvents [8]. The unique structure and physicochemical properties of BP are conducive to its application in bone tissue engineering [9]. BP can be degraded to phosphate in the presence of oxygen and water. Phosphate promotes local mineralization by capturing free Ca²⁺ [10]. BP’s electrical conductivity and biomineralization capability can improve the bone repair microenvironment [11,12]. The near-infrared (NIR) light responsiveness of BP renders it effective for photothermal/photodynamic antibacterial and antitumor applications [13]. In addition, the unique layered structure of BPNs provides them with a high specific surface area. This is advantageous for drug loading and delivery [14].

**Figure 1:**
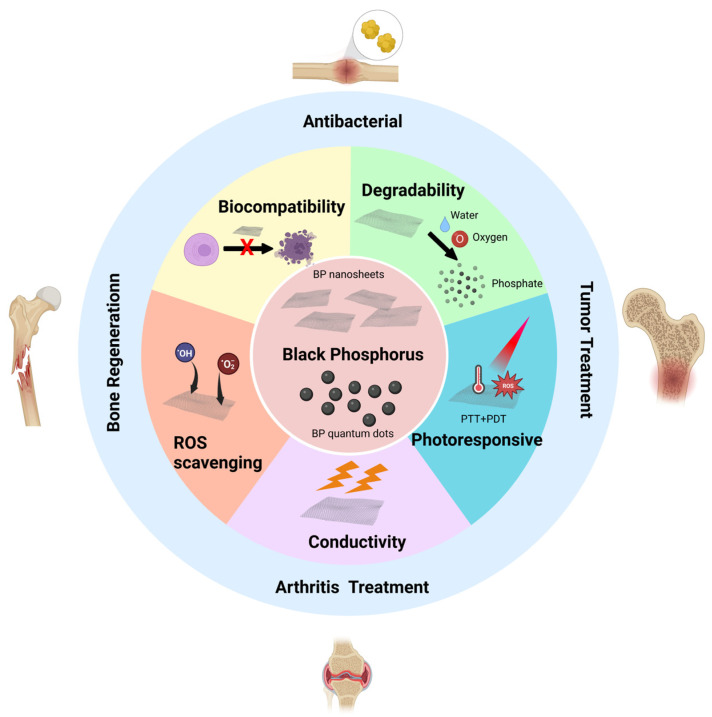
Properties of black phosphorus (BP) and its current application in bone tissue engineering. BP has good biocompatibility, degradability, antioxidant, photoresponsivity, and electrical conductivity. It has been studied in bone regeneration, infectious bone defect repair, tumor bone defect repair, and rheumatoid arthritis treatment. Developed with BioRender.com.

BP has been used to treat bone defects, bone infections, arthritis, osteosarcoma, and other diseases, and remarkable results have been achieved [15,16,17,18]. However, the large-scale application of BP in bone tissue engineering still faces challenges, such as the rapid degradation of BP, which may affect its therapeutic effect [19]. In addition, the immune response induced by BP should be fully understood. It has been reported that BP has a pro-inflammatory effect and that macrophages exposed to BP express higher levels of interferon-γ [20,21]. Currently, the application of BP in the field of biomedicine is in its infancy. Given the excellent characteristics of BP, we believe that it will provide new methods and opportunities for biomedical and orthopedic clinical treatment through the efforts of researchers. Herein, we describe the characteristics of BP and summarize the current research and possible challenges with regard to BP in bone tissue engineering. The objective is to motivate researchers (Figure 1). We used the following keywords to search the Web of Science and PubMed databases: black phosphorus AND bone OR bone regeneration OR bone tissue engineering OR arthritis OR osteomyelitis OR osteosarcoma.

## 2. Materials and Method

We used the following search terms in the Web of Science and PubMed databases for a comprehensive search of the literature published till November 2022: black phosphorus AND (bone OR bone regeneration OR bone tissue engineering OR arthritis OR osteomyelitis OR osteosarcoma). The literature related to the theme of this review was collected through literature screening.

## 3. Structure and Properties of Black Phosphorus

### 3.1. Structure of Black Phosphorus

BPNs are composed of a unique layered phosphorus structure. The layers are connected by van der Waals forces. This weak interaction force allows BP to easily peel off the surface of bulk BP crystals through different peels to yield single-layer 2D nanosheets [7,22]. Currently, the most commonly used peeling methods are liquid-phase and mechanical peeling. Other methods, such as electrochemical peeling and plasma etching, are less frequently used [23]. In BPNs, each phosphorus atom is connected to three other phosphorus atoms by covalent bonds. Furthermore, they do not exist in the same plane but in a wall-like folded lattice structure. This structure gives BP a high specific surface area and a higher drug-loading capacity than other sheet-like structured materials (which provides significant advantages for BPNs as drug transport nanocarriers) [24,25]. In 2015, Zhang et al., prepared zero-dimensional BP quantum dots (BPQDs) using liquid-phase ultrasound technology [26]. BPQDs have a higher specific surface area, smaller size, and higher bandgap. These are conducive to surface modification and functionalization [27]. In addition, BP is negatively charged because of the presence of lone pair electrons on its surface, and positively charged drugs can be loaded with BP through electrostatic interactions [28]. The modification of BP with various functional molecules or polymers can control its degradability and biocompatibility. For example, modifying BP with positively charged polyethylene glycol (PEG) can effectively improve its stability. As a result, the material has a better cancer-killing ability when the chemotherapeutic drug doxorubicin is loaded with PEG [29].

### 3.2. Biocompatibility of Black Phosphorus

Good biocompatibility is a fundamental requirement of biomedical materials. Generally, the biocompatibility of 2D nanomaterials is related to their type, size, and dosage. BP is composed of phosphorus, an essential element in the human body [1]. The degradation product of BP is phosphate, a basic component of the human body. Excessive phosphate can be excreted through the urinary system without causing any severe adverse effects. These characteristics make BP more biocompatible [30,31]. It has been reported that BPNs can exhibit cytotoxicity at concentrations greater than 50 μg mL^−1^. In contrast, BPQDs with smaller sizes (<10 nm) did not show significant cytotoxicity at concentrations up to 1000 μg mL^−1^ [32]. The cytotoxicity of BPNs is related to the size of the nanosheets, with larger BPNs (lateral dimension 884.0 ± 102.2 nm, thickness 91.9 ± 32.0 nm) being more cytotoxic than smaller ones (lateral dimension 208.5 ± 46.9 nm, thickness 17.4 ± 9.1 nm) [33]. Overall, BP is safe and biocompatible and meets the requirements of biomedical applications.

### 3.3. Degradability of Black Phosphorus

Compared with other 2D nanomaterials, the degradable properties of BPNs make them safer and give them broader prospects for biomedical applications [34]. The lone pair of electrons on the surface of BP reacts with phosphate, phosphite, and other PxOy molecules in the presence of oxygen. The moisture in the environment can remove the reacted PxOy. This exposes unreacted BP in the lower layer to form a cycle until BP is completely degraded [35,36,37]. The instability caused by degradability is one of the problems that limit the practical application of BP. In complex physiological environments, the degradation rate of BP is accelerated, and BP may be partially degraded before implantation. These problems may weaken the therapeutic effects of BP [38]. Various methods have been developed to improve the stability of BP. These include the addition of protective layers, surface chemical modification, and doping with other elements [39,40,41]. Zeng et al. modified BP using polydopamine (PDA). The encapsulation of PDA blocked oxygen and moisture from BP improves the stability of BP to a certain extent and enables it to exhibit robust photothermal properties [42]. Wang et al. used the tripeptide Fmoc-Lys-Lys-Phe (Fmoc-KKF) to modify the surface of BPNs and construct a BP@FKK complex. The modification of Fmoc-KKF improved the stability of BP, and BP@FKK exhibited better biocompatibility and cellular uptake ability [43]. Liang et al. prepared red-blood-cell-membrane-coated BPQD. The coating of the cell membrane reduces the contact of BPQD with the surrounding environment, effectively slows down the degradation of BPQD, and helps maintain the photothermal properties [44]. Deng et al. constructed BPNs that were covalently modified with superparamagnetic ferrous selenide. Ferrous selenide occupies the lone pair electrons on the surface of BP. Thereby, it reduces the exposure of lone pair electrons in the environment and enhances the stability of BP [45].

### 3.4. Photoresponsivity of Black Phosphorus

The wide tunable range of the BP bandgap enables BP to have a wide light absorption range, resulting in better optical properties than other 2D materials [46,47]. The photoresponsivity of BP has important applications in biomedicine. It enables BP to be effective in biosensing, photoacoustic imaging, photodynamic therapy, and photothermal therapy [16,48,49]. BP exhibits excellent photothermal ability under NIR light irradiation, with a photothermal conversion efficiency of 28.4% [50]. BP also exhibits a photodynamic effect and can generate a large amount of singlet oxygen under NIR light irradiation, with a quantum yield of 9.1 [51]. In recent years, researchers have widely used the photothermal and photodynamic effects of BP in antitumor and anti-infective treatments, such as combining BPQD with PLGA and loading docetaxel for a controllable chemo-photothermal antitumor therapy to achieve the synergistic induction of cancer cell apoptosis [52]. Sutrisno et al. prepared a composite scaffold of BPNs and gelatin. The concentration of BPNs determined the photothermal ability of the scaffold. A composite scaffold containing a large amount of BPNS can completely kill cancer cells in vitro and in vivo under laser irradiation [53]. Zhao et al. prepared cancer-cell-membrane-coated biomimetic BPQDs for tumor-targeted photothermal therapy combined with anti-PD-L1 immunotherapy to achieve effective anticancer therapy [54]. Huang et al. encapsulated BPQD in hydrogels as a light-responsive antibacterial platform for drug-resistant bacteria. The temperature of the platform can be elevated to 55 °C and generate reactive oxygen species (ROS) under NIR irradiation to achieve synergistic antidrug bacterial treatment [55].

### 3.5. The Oxidative Stress Regulation Ability of Black Phosphorus

ROS are important signaling molecules in cells. Maintaining a normal level of ROS is necessary for normal physiological functions [56]. However, in the presence of imbalanced ROS regulation, oxidative stress caused by high levels of ROS exerts detrimental effects on tissue repair [57]. Elevated intracellular levels of transition metal ions, such as copper ions, can cause cellular oxidative stress [58]. BP can regulate the level of copper ions in tissues by combining them with the copper ions. Thereby, it reduces ROS to playing a role in regulating oxidative stress [59]. In addition, BP exerts antioxidant effects by directly consuming ROS. Hou et al., used BP for ROS-induced acute kidney injury. The BP accumulated in the kidney effectively addressed AKI by consuming ROS [60]. Wang et al. used urokinase-loaded BPNs to treat ischemic strokes. BPNs play a neuroprotective role by reducing ROS and attenuating oxidative stress-induced reperfusion injury [61]. However, the cellular ingestion of BP can also induce oxidative stress. Mu et al. reported that BPQD can induce a large amount of intracellular ROS by inducing lipid peroxidation and reducing catalase activity [62]. When the concentration of BPQD reached 200 μg mL^−1^, the cell viability decreased significantly. Given the two-sided nature of the antioxidant effect of BP, regulating the local dose and release of BP may be one way to control its antioxidant capacity.

### 3.6. Conductivity of Black Phosphorus

Bioelectrical activity is an inherent property of human tissues. Various tissues in the human body exhibit bioelectrical conductivity or electrical sensitivity [63]. Improving the local bioelectrical activity of tissues using biomaterials is important for tissue repair [64,65]. BP has excellent electrical conductivity as a semiconductor material with an electron mobility of 1000 cm^2^ V^−1^ s^−1^ at room temperature [66]. BP can be used to improve the electrical conductivity of materials and restore local tissue electrical activity and thereby repair tissues. Qian et al. constructed BP-loaded nanoscaffolds with excellent electrical conductivity to induce angiogenesis and neurogenesis and promote myelination under mild oxidative stress conditions [12].

## 4. The Advantages of Black Phosphorus in Bone Tissue Engineering

**Figure 2:**
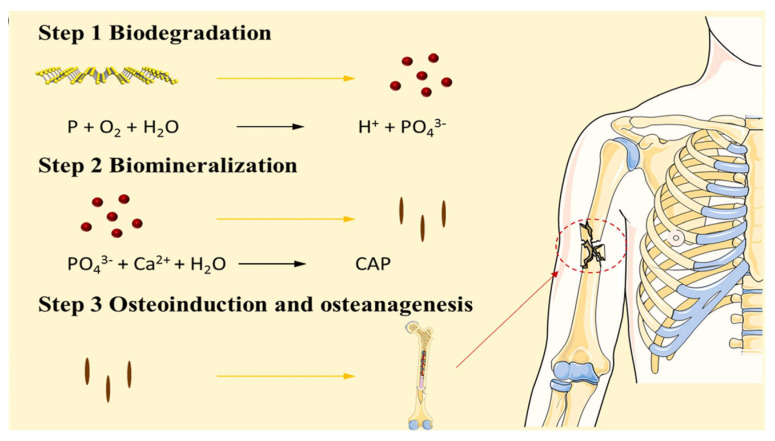
Black phosphorus degrades to phosphate in the presence of oxygen and water. Phosphate can capture calcium ions in the microenvironment and form biomineralization locally. Reproduced with permission from [72], Colloids and Surfaces B−Biointerfaces, 2022.

BP is a form of phosphorus that has a significant presence in bone tissue. Therefore, it is advantageous to apply BP in bone tissue engineering [67]. The degradability of BP prevents the potential toxicity of local accumulation [68]. Phosphate formed after BP degradation can combine with free calcium ions in the surrounding environment to form local deposits and mineralization. This promotes in situ bone tissue repair (Figure 2) [10]. Huang et al. cocultured human dental pulp stem cells with BP-loaded hydrogels. They found that BP promoted the expression of the osteogenic differentiation-related proteins Col-1, BMP4, and RUNX2 in dental pulp stem cells. This indicates that BP can promote the induction of osteogenic differentiation [10]. NIR light can accelerate the degradation rate of BP. Thereby, it regulates its degradation and the reaction between phosphate and calcium ions to accelerate the formation of local mineralization. Changing the NIR irradiation in the dimensions of time and space can play an active and controllable role in in situ mineralization, mechanical property regulation, and bone induction promotion. This is conducive to the control of the mechanical properties and osteogenic activity of biomaterials and provides a new concept for the biomedical application of BP [18,69]. Elevated phosphate concentrations in the local microenvironment directly affect osteogenic mineralization. Tang et al. studied the effects of intracellular calcium ions and phosphates on biomineralization [70]. Endoplasmic reticulum phosphate levels are elevated after exogenous phosphate is phagocytosed by the cells. Calcium ions and phosphates in the ER form calcium and phosphorus colonies and are transported to the mitochondria to form nanoscale bioluminescent precursors. These are then transported outside the cell to initiate cell-mediated bioluminescence. The size of the BPN also influences osteogenic induction. Xu et al. studied the osteogenic induction ability of BPNs of different sizes. They found that the composite hydrogel with smaller BPNs had the best osteogenic induction ability [71]. The authors proposed that this phenomenon may be because smaller BPNs have a higher specific surface area, which increases their exposure to the surrounding environment and thereby accelerates the degradation of BP and produces phosphate faster.

## 5. Research Progress of Black Phosphorus in Bone Tissue Engineering

The excellent physical and chemical properties of black phosphorus lay a good foundation for its application in bone tissue engineering. Here, we briefly summarized the application of black phosphorus in bone tissue (Table 1).

### 5.1. Bone Regeneration Materials Based on the Biomineralization Function of Black Phosphorus

The application of BP in bone tissue engineering is increasing, and many related studies have been conducted [86]. The phosphate released from the degradation of BP can undergo local biomineralization. This has a positive effect on bone regeneration. Huang et al. loaded BPNs into gelatin methacryloyl (GelMA) hydrogels for the stable release of phosphoric acid to accelerate local biomineralization [10]. BPNs improved the mechanical properties of hydrogels and released phosphoric acid in response to light to facilitate localized mineralization. The hydrogel promoted the expression of osteogenesis-related proteins and accelerated repair in a rabbit bone defect model. Xu et al. loaded BPNs into oligo[poly(ethylene glycol) fumarate] (OPF) hydrogels to prepare polymer scaffolds for bone tissue repair [73]. The composite scaffold exhibited a controllable degradation rate and phosphate release capacity. The loading of BP enhanced the adhesion, proliferation, and osteogenic differentiation of preosteogenic cells on the hydrogel surface. Jeon et al. investigated the effect of BP-coated topographical microenvironments on the cellular behavior of osteogenic precursor cells [87]. The authors prepared a BP coating on a glass substrate, which provided good supportive matrices for osteogenic precursor cells, was suitable for the survival and growth of osteogenic precursor cells and promoted the expression of osteogenesis-related genes. We believe that modifying bioinert surfaces with BP coatings can improve the biocompatibility and osteogenic activity of materials.

**Figure 3:**
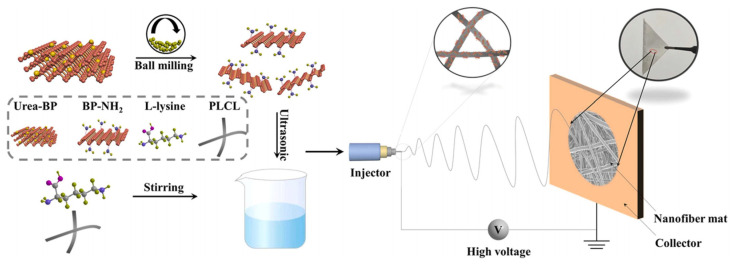
The PLCL/L-NH-BP nanocomposite scaffold prepared by electrospinning can promote the osteogenic differentiation of mesenchymal stem cells. Reproduced with permission from [72], Colloids and Surfaces B-Biointerfaces, 2022.

On the one hand, the degradability of BP can promote osteogenesis. On the other hand, excessive degradation may affect its bone repair effect [88]. By modifying BP, its stability can be improved to a certain extent, and additional functions can be added [9]. Wang et al. loaded PDA-modified BPNs into poly l-lactic acid (PLLA) scaffolds to improve their mechanical properties and induce in situ biomineralization [74]. PDA exhibits good biocompatibility and stability. In a weakly alkaline environment, dopamine spontaneously formed a PDA layer on the surface of a substrate through rearrangement and crosslinking to wrap the substrate [89,90]. The stability of the BPN improved because of the coating of the PDA layer, and the addition of PDA@BP to the PLLA scaffold improved the mechanical properties of the scaffold. After the PLLA/BP@PDA scaffolds were immersed in a simulated body fluid (SBF) solution for 14 d, a large number of calcium phosphate crystals were formed on the surface of the scaffolds. Compared with PLLA scaffolds, the in situ mineralization ability of PLLA scaffolds was significantly improved. This has a positive effect on osteogenic differentiation [91]. In another report, researchers loaded lysine-modified BP onto L-lactate (L-NH-BP) and caprolactone poly-(lactide-coε-caprolactone) (PLCL) electrospun scaffolds to construct biodegradable scaffolds with good biocompatibility (Figure 3) [72]. The scaffolds promoted the proliferation and osteogenic differentiation of mesenchymal in vitro.

**Figure 4:**
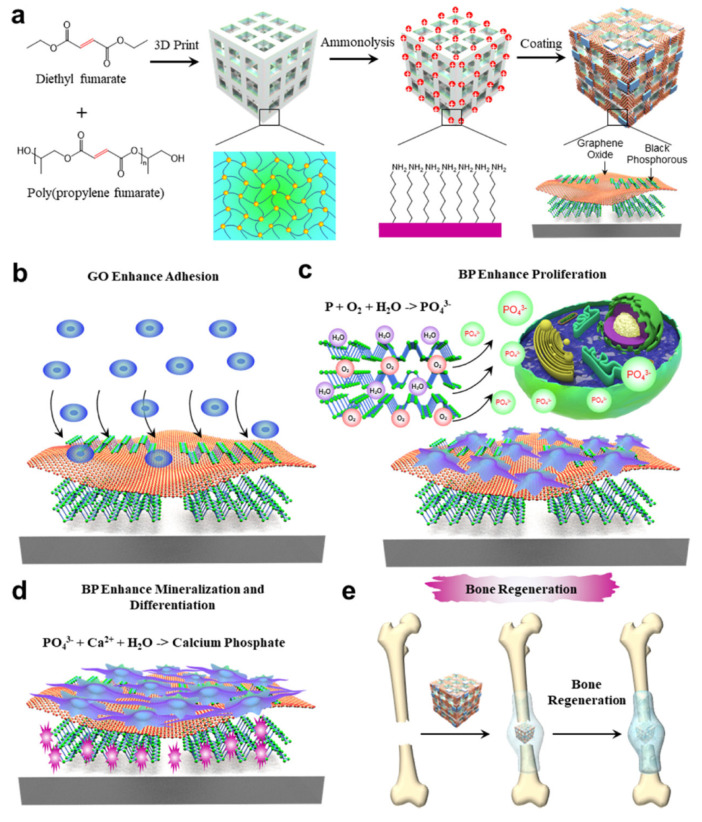
(**a**) Constructing dual two−dimensional nanomaterial (GO@BP) co−functionalized polypropylene fumarate (PPF) three−dimensional (3D) scaffold by using graphene oxide (GO) and black phosphorus nanosheets (BPNs). (**b**) The loading of GO enhanced the ability of cells to adhere to the scaffold surface. (**c**) The degradation of BPNs released phosphate to promote cell proliferation. (**d**) The calcium phosphate formed locally by phosphate and calcium ions improved the biomineralization function of the scaffold. (**e**) The repair process of GO@BP co−functionalized 3D scaffold for bone defects. Reproduced with permission from [75], Chemical Engineering Journal, 2022.

An important strategy for promoting bone repair is to adjust the mechanical properties, chemical composition, and biological signals of hydrogels to simulate the extracellular matrix (ECM) microenvironment of bone regeneration [92]. Wang et al. constructed a dual-network hydrogel loaded with BPNs to simulate the ECM microenvironment during bone repair [93]. The authors added methacrylate-modified gelatin, polysaccharide, and BPNs with mineralized CaP crystal carriers to the hydrogels to simulate collagen, polysaccharide, and the mineralized crystallization of the natural bone ECM. The addition of BP induced rapid biological mineralization in the hydrogels and promoted osteogenic activity in the cells. Mesenchymal stem cell spheroids have attracted attention because of their advantages in cell–cell and cell–extracellular matrix interactions [94]. Li et al. constructed mesenchymal stem cell spheroids containing collagen and BPNs. These showed excellent cytocompatibility and osteogenic ability after 14 days of culture in osteogenic medium [95]. The authors loaded spheres containing 6 μg mL^−1^ collagen and 4 μg mL^−1^ BP in the OPF hydrogel that had better osteogenic capacity than the control group. Liu et al. used graphene oxide (GO) and BPNs to construct dual 2D nanomaterial GO@BP co-functionalized polypropylene fumarate (PPF) three-dimensional (3D) scaffolds (Figure 4) [75]. GO has a large surface area and strong protein adsorption capacity. These are conducive to cell adhesion and proliferation [96,97]. The loading of GO@BP in 3D-printed scaffolds enhanced the adsorption capacity of scaffold proteins to promote cell adhesion. Subsequently, phosphate released by BP degradation played an osteogenic-promoting role. In another report, the authors fabricated an injectable bone repair material containing carbon nanotubes and BP loaded into an OPF hydrogel [98]. In addition to promoting osteogenic repair through the phosphate degradation of BP, electrical stimulation based on hydrogel application positively affected bone repair. The hydrogel accelerated bone defect repair in a rabbit model of femoral defect and vertebral fusion. Tan et al. loaded chitosan/collagen-based hydrogels with BP coated with mesenchymal stem cell membranes to simulate phosphate ions enriched in the bone ECM [99]. Membrane-coated BPNs can generate mild heat and activate heat shock proteins to recruit osteoblasts. Phosphate released by BP degradation can capture calcium ions and form hydroxyapatite in the ECM. This is conducive to the migration and differentiation of osteoblasts.

### 5.2. Vascularized Bone Regeneration Material Based on Black Phosphorus

**Figure 5:**
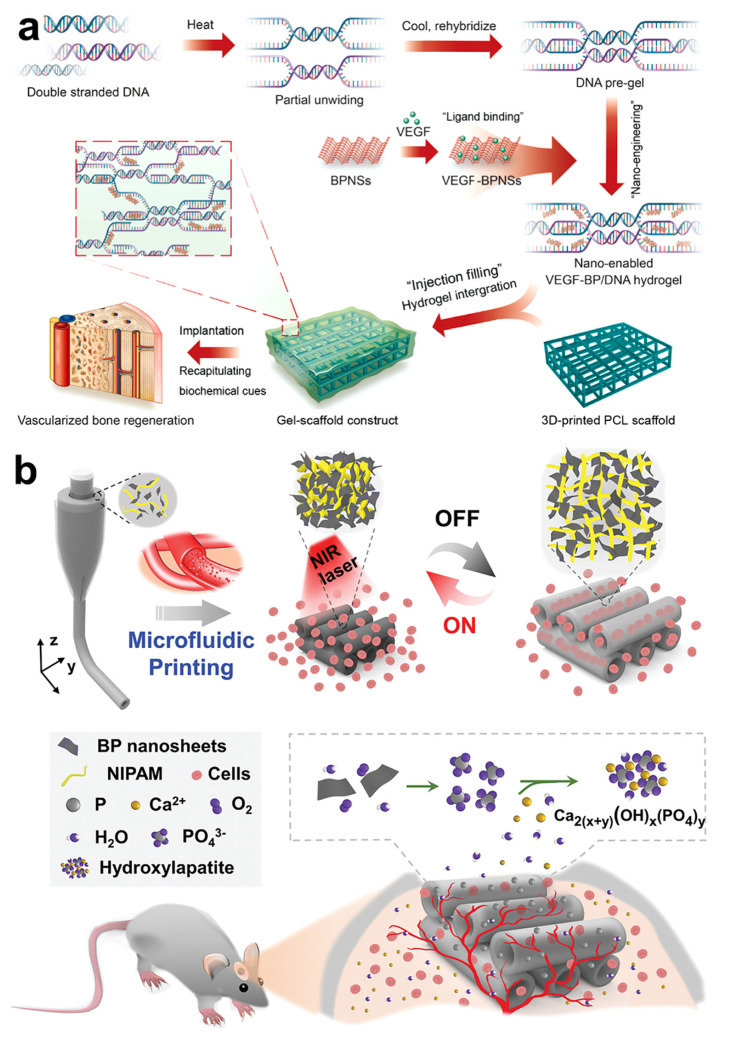
(**a**) DNA−PCL scaffolds loaded with vascular endothelial growth factor (VEGF)−modified black phosphorus (BP) nanosheets (BPNs) achieve vascularized bone regeneration by releasing VEGF; reproduced with permission from [77], Bioactive Materials, 2023. (**b**) Microfluidic three−dimensional printed scaffolds containing BPNs can achieve reversible contraction and expansion of the scaffold under the irradiation of near−infrared light. Thereby, these promote the penetration of vascular endothelial cells into the scaffold and induce vascularized bone regeneration. Reproduced with permission from [105], Advanced Functional Materials, 2021.

Angiogenesis is an important process for bone tissue repair. Neovascularization can provide nutrients and oxygen to bone defects and secrete bioactive factors during new bone formation [100,101]. The co-delivery of BP and vasculogenic substances is a feasible strategy for achieving efficient vascular regeneration in bone defects. Deferoxamine is an angiogenesis promoter that stimulates the hypoxia-inducible factor 1-α pathway to promote angiogenesis by mimicking the local hypoxic environment [102,103]. Xu et al. developed a GelMA hydrogel co-doped with deferoxamine and BP to repair ischemic bone defects [76]. The hydrogel promoted the local expression of CD31 and positively affected vascular repair. The co-delivery of BP and deferoxamine exerted a synergistic effect on osteogenic repair. Miao et al. applied vascular endothelial growth factor (VEGF)-modified BPNs to DNA hydrogels to promote vascularized bone repair (Figure 5a) [77]. BP enhances the mechanical strength of the hydrogel. The continuous release of VEGF bound to BPNs through noncovalent interactions in the hydrogel accelerates vascular regeneration. In vivo experiments showed that the hydrogel exhibited synergistic angiogenic and osteogenic effects. Nerves play a leading role in bone defect repair as they are the first to grow into bone defect sites and promote vascular regeneration [104]. The electrical conductivity of BP promotes nerve repair. Furthermore, it has been previously reported that BP contributes to peripheral nerve repair and vascular regeneration [12]. Xu et al. modified BP with magnesium ions and loaded it into a double-layer scaffold to promote neurovascularized bone regeneration [78]. The upper hydrogel was used to simulate the periosteum structure, in which the loaded magnesium-modified BPN could release magnesium ions to play a role in vascular regeneration and nerve repair. The results of in vivo experiments showed that the hydrogel scaffold promoted early nerve regeneration and angiogenesis. This was beneficial for the rapid repair of bone tissue. Wang et al. developed a microfluidic scaffold based on BP with reversible heat-responsive contraction and expansion behaviors (Figure 5b) [105]. Under the photothermal effect generated by BP, N-isopropylacrylamide in the scaffold underwent a temperature-induced volumetric phase transition. This triggered reversible contraction and expansion behaviors to promote the entry of the suspension cells into the scaffold. The vascular regeneration ability of the scaffolds was investigated. The photothermal response behavior of the scaffold promoted the growth of vascular endothelial cells in the scaffold. This was conducive to vascularized bone regeneration.

### 5.3. Bone Regeneration Material Based on Photothermal Responsiveness of Black Phosphorus

Mild thermal stimulation can promote the repair of bone defects [106]. The heat generated by NIR light can be regulated by adjusting the intensity of the light source and content of the photothermal-responsive agent loaded in the material. Local thermotherapy using the high-efficiency photothermal conversion ability of BP can also effectively promote bone tissue repair. It has been reported that local photothermal therapy using BP can upregulate the expression of type 1 collagen (Col-1) and heat shock proteins and accelerate the process of bone tissue repair [19]. Chen et al. loaded BP, Sr, and ibuprofen into sodium alginate microspheres and loaded them onto PLLA nanofiber scaffolds [79]. BP enhanced the photothermal response of the scaffold. Furthermore, the controlled release of Sr and ibuprofen was achieved under NIR irradiation. In vitro experiments showed that the scaffold had good cell adhesion and proliferation and could induce osteogenesis by inducing in situ biomineralization. Matrix vesicles (MVs) are extracellular vesicles released by bone-related functional cells that play an essential role in the regulation of biomineralization [107]. Previous studies have reported that increasing the phosphate concentration in and around the MVs can accelerate bone tissue repair [108]. Wang et al. embedded BP in MVs and modified MVs with aptamers to enhance biomineralization and osteoblast-targeting ability [109]. After the targeted delivery of MV by the aptamer, the photothermal response ability of BP can be used to stimulate the expression of heat shock proteins and alkaline phosphatases to promote osteogenic repair. Li et al. used microfluidic technology to develop a fibrous scaffold that could provide bioactive elements to bone-defect areas [80]. BP, hydroxyapatite, and silica loaded on the fibrous scaffold released phosphate, calcium, and silicon ions in the defect area to promote osteogenesis. In addition, the photothermal response of the scaffold promoted ion release, which has a positive effect on bone regeneration [110]. Wang et al. developed a light-triggered drug-delivery platform by loading strontium chloride and BPNs into PLGA microspheres [18]. The microspheres exhibited excellent cell compatibility and degradation ability. Under NIR irradiation, the heat generated by BP accelerated the degradation of PLGA microspheres and promoted the release of strontium ions, thereby achieving controlled drug delivery. The authors used microspheres to treat femoral defects in rats. The microspheres showed a good ability to promote bone repair under NIR light irradiation.

Irregular bone defects are a problem in clinical orthopedic treatment. For such bone defects, customized 3D-printed scaffolds cannot be directly implanted into the defect site. This imposes additional surgical burden [111]. Wang et al. added a β-tricalcium phosphate/poly(lactic acid-co-trimethylene carbonate) (TCP/P(DLLA-TMC)) nanocomposite with BP as a photothermal agent scaffold to construct a four-dimensional-printed scaffold with shape memory function [112]. When the scaffold was heated to 45 °C by NIR light irradiation, the shape reconfiguration of the scaffold occurred to facilitate the implantation of the scaffold in irregular bone defects. The implanted scaffolds had good mechanical properties at 37 °C and could slowly release osteogenic active peptides to promote bone defect repair.

### 5.4. Antibacterial Bone Regeneration Material Based on Black Phosphorus

Inspired by the chloroplast structure, Zhao et al. developed an antibacterial osteogenic scaffold based on BP and silver nanoparticles to repair infected bone defects [113]. BPNs protected by PDA and linked with silver were loaded onto a 3D porous chitosan and polycaprolactam composite fiber network to construct a biological platform for chloroplast simulation. The scaffold showed a strong antibacterial effect. Furthermore, the mild heat generated by NIR light irradiation effectively promoted the expression of osteogenesis-related proteins and accelerated the repair of the infected bone defects. Miao et al. prepared a photothermal antibacterial therapeutic nanocomposite hydrogel (BP/gel) by loading BPNs into GelMA hydrogel [81]. BPNs enhanced the cross-linking network and mineralization ability of the hydrogels. Under NIR light irradiation, the hydrogel could be heated up to 55.3 °C, thereby showing efficient antibacterial and antitumor effects in vitro. Additionally, the osteogenic induction ability of the hydrogels was verified. The addition of BP enabled the hydrogel to promote bone formation in vitro without osteogenic factors. This promoted bone defect healing in Sprague–Dawley (SD) rat skull defects. Wu et al. prepared a photothermal antibacterial scaffold by loading BPNs modified with a zinc sulfonate ligand onto the surface of a hydroxyapatite scaffold [15]. BP and zinc sulfonate ligands have a synergistic antibacterial effect, whereby the scaffold can exert an antibacterial effect below 50 °C. This minimizes the tissue damage caused by high temperatures. After anti-infective treatment, mild photothermal at 40–42 °C combined with zinc ions and phosphate released from the scaffold exerted a synergistic osteopromoting effect. Qing et al. constructed a multifunctional hydrogel with photothermal antibacterial and bioactive ion-release properties using a poly (vinyl alcohol)/chitosan hydrogel loaded with magnesium oxide nanoparticles and BPNs [114]. The photothermal effect of BP and the antibacterial ability of chitosan play a synergistic role. The magnesium and phosphate ions released by the hydrogel can promote the biomineralization, migration, and osteogenic differentiation of mesenchymal stem cell (MSC) to accelerate the repair of bone defects [115].

**Figure 6:**
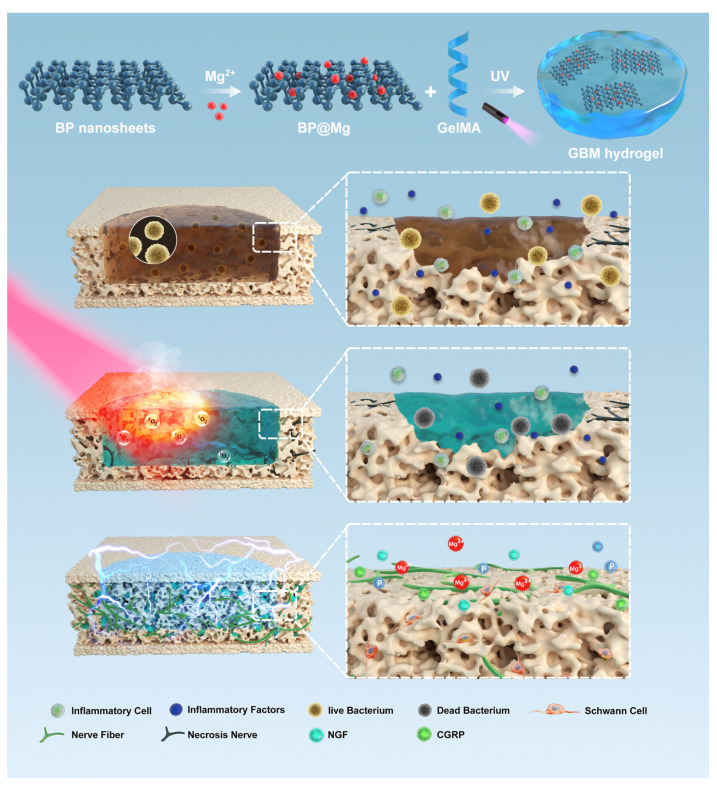
Schematic of GelMA-BP@Mg (GBM) hydrogel for antibacterial and innerved bone regeneration of infected bone defects. Under NIR irradiation, GBM hydrogel displays a high antibacterial efficiency and reduces the damage to bone tissue by bacteria. Magnesium-modified black phosphorus nanosheets promote skeletal-associated nerve fiber repair and accelerate bone regeneration. Reproduced with permission from [83], Advanced Healthcare Materials, 2022.

Sensory nerve fibers play an important role in bone repair [104]. As previously reported, nerve repair peaks on the first day of bone repair and influences the subsequent vascularization, osteogenic differentiation, and mineralization [116]. Bone infections cause an unfavorable microenvironment around the defect area. Excessive inflammatory factors and acidic substances in the microenvironment caused by bacteria and inflammatory cells are not conducive to nerve repair and may affect the repair of bone defects [117,118]. Jing et al. loaded magnesium-modified BPNs into GelMA to develop a photosensitive conductive hydrogel for the repair of infected bone defects [83] (Figure 6). The hydrogel can be heated to over 50 °C under NIR irradiation of 1 W/cm^2^. It displays a high antibacterial effect in vitro and in vivo. The continued release of magnesium ions promotes Schwann cells to secrete more nerve growth factors. Combined with the electrical conductivity of the hydrogel, this induces further nerve growth in bone defects. The hydrogel effectively promoted intrinsic bone regeneration in a model of infected bone defects.

One of the challenges in treating infected bone defects is the difficult-to-cure infections caused by biofilms formed by microbial infections [119]. Coating the surface of implants to improve their osteoinductive activity while acting as an anti-biofilm is a feasible research direction for infected bone defects [120]. Yuan et al. prepared a layer of BPNs and hydroxyapatite composite coating on the surface of metal implants to treat bone infections [82]. The coating had excellent photothermal conversion ability (heating up to 60 °C after 1 W/cm^2^ NIR light irradiation) and good anti-biofilm properties. In addition, BP and hydroxyapatite exhibited a synergistic bone-repair effect to promote the healing of infected fractures.

### 5.5. Antitumor Bone Regeneration Materials Based on Black Phosphorus

**Figure 7:**
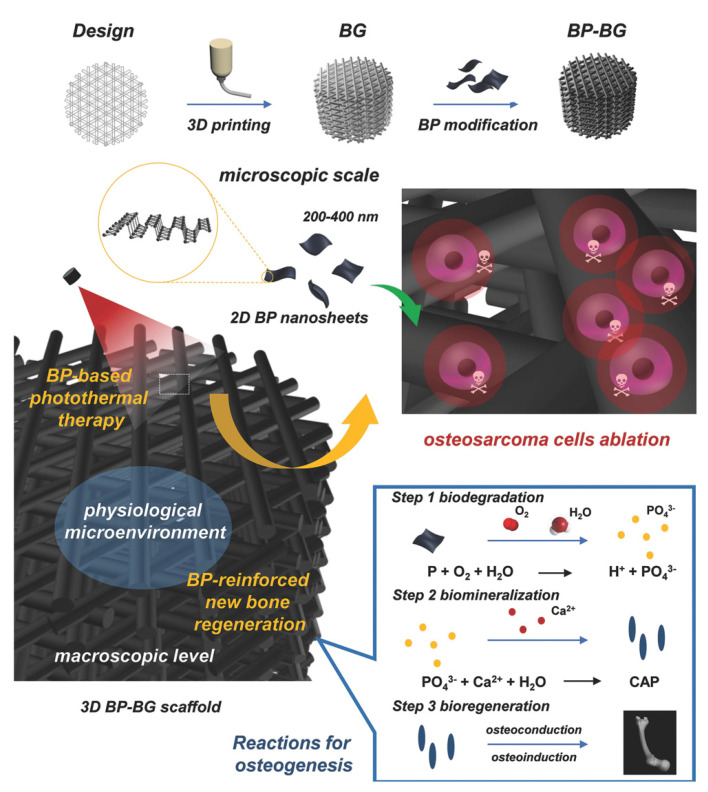
Preparation of black phosphorus−bioactive glass scaffold and its stepwise treatment strategy for osteosarcoma. Reproduced with permission from [84], Advanced Materials, 2018.

The photothermal and photodynamic effects of BP can effectively kill tumor cells. This improves the microenvironment and promotes bone tissue repair. Yang et al. prepared a dual-functional scaffold with photothermal antitumor and osteogenic repair promotion ability by loading BPNs into 3D-printed biogas scaffolds (Figure 7) [84]. The scaffold had an excellent photothermal response and could be heated up to 68.7 °C after 5 min of 1 W/cm^2^ NIR light irradiation. This met the requirements of photothermal antitumor. Bioactive glass scaffolds are conducive to cell proliferation, differentiation, and angiogenesis and can synergize with BP to promote osteogenic repair. Wang et al. developed a low-temperature 3D-printed polymer scaffold loaded with doxorubicin hydrochloride, β-tricalcium phosphate, BPNs, and osteogenic peptide P24 for treating tumoral bone defects [13]. The photothermal function of the stent based on BP is used for on-demand photothermal tumor killing after implantation and the possibility of preventing local tumor recurrence by continuously releasing low concentrations of doxorubicin hydrochloride. In addition, the presence of TCP and osteogenesis-related peptides in the scaffold can have a continuous osteogenic effect that improves the repair of bone defects. Ma et al. constructed a BP/doxorubicin hydrochloride/PDA coating on a NiTi alloy surface to treat bone tumors and bone defects [85]. The authors fabricated groove-like microstructures and nanostructures on the surface of a NiTi alloy using a femtosecond laser to promote osseointegration of the implants. Then, they coated the surface with BP/doxorubicin hydrochloride/PDA to achieve NIR light/pH dual control of antitumor drug release. In addition, the antibacterial properties of the implant were tested. It showed high antibacterial activity against both *Staphylococcus aureus* and *Pseudomonas aeruginosa* under NIR light irradiation.

### 5.6. Applications of Black Phosphorus-Based Biomaterials in Other Orthopedic Diseases

Rheumatoid arthritis is characterized by the excessive proliferation of synovial cells, which destroy cartilaginous joints and bone tissue. This requires treatment to remove excess synovial tissue and repair damaged bone tissue. Pan et al. developed a chitosan/platelet-rich plasma hydrogel loaded with BPNs to treat rheumatoid arthritis [17]. After the hydrogel is injected into the joint cavity, the photothermal and photodynamic effects of BP can be used to remove the excess synovial tissue. In addition, phosphate released from the hydrogel can subsequently promote bone tissue repair. This makes hydrogel suitable for treating rheumatoid arthritis. Sun et al. used polyetheretherketone (PEEK)/polytetrafluoroethylene and BPNs to construct an artificial joint material with antibacterial properties and wear resistance [121]. The wear rate of the composite material was significantly improved compared with that of pure PEEK. Furthermore, the addition of BP resulted in antibacterial properties, with an antibacterial rate of 99.9%.

## 6. Conclusions and Outlook

Since the successful preparation of BPNs in 2014, research on BP in biomedicine has received considerable attention. The excellent characteristics of BP make it the basis for extensive tissue engineering research. Research on BPNs and BPQD has been reported in many fields, including biosensing, drug delivery, cancer treatment, and tissue repair [16,122,123,124]. This review summarizes the structure and properties of BP and its advantages in bone tissue engineering. In addition, it comprehensively summarizes the current research and application progress of BP in bone tissue engineering. The main application strategies of BP in bone tissue engineering include the following: (1) The degradation of BP into phosphate in the presence of oxygen and water can capture calcium ions in the surrounding environment to form local mineralization to simulate the mineralized components in bone tissue and promote bone formation. (2) Utilizing the photothermal conversion ability of BP, NIR light is used to generate mild heat locally, activating the expression of local tissue heat shock proteins, alkaline phosphatase, and other osteogenesis-related proteins to promote bone repair. It can also exert antibacterial, antitumor, and synovial cell clearance effects through high heat and photodynamic effects to improve the local pathological microenvironment and promote bone regeneration. (3) The high specific surface area of BP is used as a drug delivery carrier to deliver bioactive ions and biological factors to local bone defects to play a role in osteogenesis. These strategies have been applied in bone tissue engineering and have achieved specific expected effects, thus laying a foundation for the subsequent improvement of treatment strategies [86].

One of the current research directions for BP is to improve its original function or obtain functions that are unaffected by certain functional modifications. The lack of penetration depth of NIR light may limit its clinical application in orthopedics [125]. Ultrasound-driven sonodynamic therapy is an emerging cancer treatment method. Liu et al. constructed three types of functionalized BPNs using a covalent modification method. The fullerene-functionalized BPNs and benzoic acid-functionalized BPNs exhibited stronger sonodynamic effects than the BPNs and produced more hydroxyl radicals. Thus, they have good application prospects in ultrasound-driven anticancer therapy. Tan et al. used poly (4-pyridone methyl styrene) endoperoxide (PPMS-EPO) and BPNs to fabricate an antibacterial film, PPMS-EPO/BPS [126]. PPMS-EPO can store singlet oxygen, which is generated after exposure to light. In the absence of external light, this hybrid antibacterial film could release ROS to exert an antibacterial effect and improve the stability of BP. The antibacterial rates of *Escherichia coli* and *Staphylococcus aureus* without infrared light irradiation were 76.5% and 69.7%, respectively. These studies will stimulate researchers to develop new functions and applications for BP.

However, compared with other nanomaterials, such as graphene, the research on BP is still in its early stages [127,128]. Many problems still need to be solved and improved. First, BP is expensive, unstable in unprotected conditions, and prone to degradation. This limits its clinical use and may undermine the expected efficacy [129,130]. The preparation and preservation methods of BP need to be optimized further for large-scale applications. Second, the immune response elicited by BP must be defined in vivo. According to previous reports, the uptake of BP by macrophages decreases its activity and promotes the secretion of inflammatory cytokines including TNF-α [131]. BP may form a protein crown upon contact with hemoglobin, affect cellular uptake, activate the NF-κB pathway, and induce an immune response [20]. The immune response induced by BP may destroy the local microenvironment, thereby affecting its tissue-repair function. The modification of BP is a feasible means of regulating inflammatory responses. The role of BP in the regulation of oxidative stress requires further investigation. Some researchers have used the degradation ability of BP to exert an antioxidant effect, which is important for improving the local tissue repair environment [60]. However, a high concentration (200 μg mL^−1^) of BPQD can induce lipid peroxidation in cells and affect cell viability [62]. Balancing the antioxidant and oxidative stress of BP and its antioxidant function in bone tissue engineering has not been reported. Currently, the research on BP is becoming increasingly extensive and in-depth. We believe that with the joint efforts of researchers, the application of BP in bone tissue engineering will be developed better. This will lay a firm foundation for large-scale clinical applications of BP and other nanomaterials in orthopedics.

## Figures and Tables

**Table 1:** Summary of the application of black phosphorus in bone tissue engineering.

Target	Material Design	Main Results	Reference
Biomineralization	BPNs-GelMA	Promoted the expression of osteogenesis-related genes and accelerated bone repair.	[10]
	BPNs-OPF	Exhibited a controllable degradation rate and phosphate release capacity. Enhanced the adhesion, proliferation, and osteogenic differentiation of pre-osteogenic cells.	[73]
	PDA@BPNs-PLLA	Improved the stability of the BPN and promoted osteogenic differentiation.	[74]
	L-NH-BP-PLCL	Promoted the proliferation and osteogenic differentiation of mesenchymal.	[72]
	GO@BP-PPF	Promoted cell adhesion and osteogenic differentiation.	[75]
Vascularized Osteogenesis	Deferoxamine/BP-GelMA	Promoted local expression of CD31 and positively affected a vascular repair.	[76]
	VEGF@BPNs-DNA hydrogel	Continuous release of VEGF. Accelerated vascular regeneration and bone regeneration.	[77]
	BP@Mg double-layer scaffold	Promoted early nerve regeneration and angiogenesis in the process of bone regeneration.	[78]
Photothermal Osteogenesis	BP/IBU@SA-PLLA	Significantly high photothermal conversion efficiency and photothermal-responsive intelligent drug release performance.	[79]
	SrCl_2_/BPNs-PLGA	Remarkable cell compatibility and degradation capability. Remarkably controlled release of strontium ions.	[18]
	BP@HA/SiO_2_-PLLA	The photothermal effect promoted the release of elements, thereby achieving accelerated osteogenesis.	[80]
Antibacterial	BPNs-GelMA	The hydrogel could be heated up to 55.3 ℃ and showed efficient antibacterial and antitumor effects.	[81]
	BPNs/HA coating	Remarkable photothermal conversion capability and good anti-biofilm properties.	[82]
	BP@Mg-GelMA	Remarkable antibacterial capability and induced innerved bone regeneration.	[83]
Antitumor	BPNs -BG scaffold	Remarkable photothermal antitumor activity and osteogenic induction ability.	[84]
	BP/doxorubicin hydrochloride/PDA coating	NIR/pH dual controlled antitumor drug release and high antibacterial activity.	[85]

## Data Availability

Not applicable.
